# Purification and Characterization of Suicin 65, a Novel Class I Type B Lantibiotic Produced by *Streptococcus suis*

**DOI:** 10.1371/journal.pone.0145854

**Published:** 2015-12-28

**Authors:** Katy Vaillancourt, Geneviève LeBel, Michel Frenette, Nahuel Fittipaldi, Marcelo Gottschalk, Daniel Grenier

**Affiliations:** 1 Groupe de Recherche en Écologie Buccale (GREB), Faculté de Médecine Dentaire, Université Laval, Quebec City, Quebec, Canada; 2 Centre de Recherche en Infectiologie Porcine et Avicole (CRIPA), Fonds de Recherche du Québec - Nature et Technologies (FQRNT), Saint-Hyacinthe, Quebec, Canada; 3 Public Health Ontario and Department of Laboratory Medicine and Pathobiology, Faculty of Medicine, University of Toronto, Toronto, Ontario, Canada; 4 Groupe de Recherche sur les Maladies Infectieuses du Porc (GREMIP), Faculté de Médecine Vétérinaire, Université de Montréal, Saint-Hyacinthe, Quebec, Canada; University of Kansas Medical Center, UNITED STATES

## Abstract

Bacteriocins are antimicrobial peptides of bacterial origin that are considered as a promising alternative to the use of conventional antibiotics. Recently, our laboratory reported the purification and characterization of two lantibiotics, suicin 90–1330 and suicin 3908, produced by the swine pathogen and zoonotic agent *Streptococcus suis* (serotype 2). In this study, a novel bacteriocin produced by *S*. *suis* has been identified and characterized. The producing strain *S*. *suis* 65 (serotype 2) was found to belong to the sequence type 28, that includes strains known to be weakly or avirulent in a mouse model. The bacteriocin, whose production was only possible following growth on solid culture medium, was purified to homogeneity by cationic exchange and reversed-phase high-pressure liquid chromatography. The bacteriocin, named suicin 65, was heat, pH and protease resistant. Suicin 65 was active against all *S*. *suis* isolates tested, including antibiotic resistant strains. Amino acid sequencing of the purified bacteriocin by Edman degradation revealed the presence of modified amino acids suggesting a lantibiotic. Using the partial sequence obtained, a blast was performed against published genomes of *S*. *suis* and allowed to identify a putative lantibiotic locus in the genome of *S*. *suis* 89–1591. From this genome, primers were designed and the gene cluster involved in the production of suicin 65 by *S*. *suis* 65 was amplified by PCR. Sequence analysis revealed the presence of ten open reading frames, including a duplicate of the structural gene. The structural genes (*sssA* and *sssA’)* of suicin 65 encodes a 25-amino acid residue leader peptide and a 26-amino acid residue mature peptide yielding an active bacteriocin with a deducted molecular mass of 3,005 Da. Mature suicin 65 showed a high degree of identity with class I type B lantibiotics (globular structure) produced by *Streptococcus pyogenes* (streptococcin FF22; 84.6%), *Streptococcus macedonicus* (macedocin ACA-DC 198; 84.6%), and *Lactococcus lactis* subsp. *lactis* (lacticin 481; 74.1%). Further studies will evaluate the ability of suicin 65 or the producing strain to prevent experimental *S*. *suis* infections in pigs.

## Introduction

*Streptococcus suis* is a major swine pathogen worldwide that is transmitted via the respiratory route and colonizes the palatine tonsils of pigs [1]. Given its ability to produce a large array of virulence factors, it can cause meningitis, septicemia, arthritis, and endocarditis [2, 3]. Although *S*. *suis* infections in humans remain sporadic and affect mainly individuals in close contact with sick or pig-derived products, important outbreaks that occurred in Asia modified the world viewpoint regarding the threat of *S*. *suis* for humans [4, 5]. While 35 serotypes (1 to 34 and 1/2) have been identified, serotype 2 is the most frequently associated with pathology [1, 2]. However, in the last decade, other serotypes with a particular geographical distribution have also been identified as the source of many infections [6]. For instance, in North America, serotypes 2 and 3 show a prevalence of 24.3% and 21%, respectively, followed by serotypes 1/2, 8, and 7 [6].

Due to the wide diversity among strains, current vaccines for *S*. *suis* infections confer only a limited and serotype-specific protection [7]. For this reason, studies are still needed to develop an effective and broadly protective vaccine against the most prevalent if not all pathogenic serotypes. Antibiotics administrated as prophylactic or methaphylactic treatment are usually effective for controlling disease progression in the herds [8]. However, antibiotics should be used carefully and adequately to reduce the selection of resistant *S*. *suis* isolates. While most strains of *S*. *suis* are sensitive to penicillin and amoxicillin, resistance to macrolides, lincosamides, sulphonamides, and tetracyclines has been reported with up to 85% of strains showing resistance [9, 10].

With the rising concern on resistance of pathogens to conventional antibiotics, bacteriocins, which are ribosomally synthesized antimicrobial peptides of bacterial origin, have been proposed as a promising alternative antimicrobial strategy in animal agriculture [11, 12]. Bacteriocins are cationic bactericidal peptides that kill bacteria by disrupting the cell membrane integrity [13]. More specifically, lantibiotics are an important family of heat stable low molecular weight bacteriocins that possess unusual post-translationally modified amino acids, such as lanthionine, methyllanthionine, dehydroalanine, and dehydrobutyrine with thioether linkages [14]. Nisin A represents the most studied lantibiotic, being used commercially as a food preservative especially in dairy products in more than 50 countries [15]. Recently, our laboratory purified and characterized two lantibiotics produced by *S*. *suis* serotype 2 [16, 17]. Suicin 90–1330 is a type A (linear) lantibiotic secreted by a non-virulent strain of *S*. *suis* that exhibits high homology with nisin U produced by *Streptococcus uberis* [16]. Suicin 3908 is a type B (N-terminal linear and C-terminal globular moieties) lantibiotic produced by a strain of *S*. *suis* isolated from an healthy carrier pig and that shows a high homology with bovicin HJ50 (*Streptococcus bovis*) and thermophilin 1277 (*Streptococcus thermophilus*) [17]. As a continuation of our ongoing work aimed to investigate *S*. *suis* bacteriocins, in this study, we identified and characterized a novel class I type B lantibiotic produced by *S*. *suis*.

## Materials and Methods

### Bacterial strains and growth conditions

Strains of *S*. *suis* used in this study are listed in Table 1. Bacteria were routinely grown aerobically under static conditions at 37°C in Todd Hewitt broth (THB; BD-Canada, Mississauga, ON, Canada).

**Table 1:** Strains of *S*. *suis* used in this study, their characteristics and susceptibility to *S*. *suis* 65.

Strain	Country	Origin	ST	Resistance to antibiotics^1^		Inhibitory zone (mm) ^2^ produced by *S*. *suis* 65
				Erythromycin	Tetracycline	
31533	France	Meningitis	1	Sensitive	Sensitive	4.7 ± 0.6
MNCM01	Thailand	Endocarditis (human)	1	Resistant	Resistant	2.0 ± 1.0
MNCM06	Thailand	Meningitis (human)	1	Resistant	Resistant	3.7 ± 0.6
P1/7	United Kingdom	Meningitis	1	Sensitive	Sensitive	4.3 ± 0.6
MGGUS3	United States	Meningitis	1	Unknown	Unknown	5.3 ± 0.5
89–1591	Canada	Septicemia/meningitis	25	Unknown	Unknown	1.2 ± 0.3
1043248	Canada	Meningitis	25	Unknown	Unknown	4.3 ± 0.6
MNCM51	Thailand	Septicemia (human)	25	Unknown	Unknown	6.0 ± 1.0
LPH4	Thailand	Septicemia (human)	25	Resistant	Resistant	5.0 ± 1.0
MGGUS4	United States	Septicemia	25	Unknown	Unknown	3.3 ± 0.6
1054471	Canada	Meningitis	28	Sensitive	Resistant	3.3 ± 0.6
1057906	Canada	Meningitis	28	Unknown	Unknown	3.7 ± 0.6
1088563	Canada	Meningitis	28	Resistant	Resistant	3.3 ± 0.6
65	France	Tonsils, healthy pig carrier	28	Unknown	Unknown	0
DAT245	Japan	Meningitis	28	Unknown	Unknown	4.0 ± 1.0
MGGUS10	United States	Pneumonia	28	Unknown	Unknown	4.5 ± 0.7

^1^ A strain was considered resistant if the MIC was ≥ 400 μg/ml

^2^ Means ± standard deviations of triplicate assays

### Plate diffusion assay for bacteriocin production

Overnight cultures of *S*. *suis* were spotted (2 μl) on Todd Hewitt agar (THA; BD-Canada) plates (100 mm) supplemented with 0.01% Tween 80 (sorbitan polyoxyethylene monooleate; Sigma-Aldrich Canada Co., Oakville, ON, Canada), which were incubated at 37°C for 24 h. Plates were then overlaid with THB soft-agar (0.75%, w/v) that had been inoculated (750 μl in 7.5 ml) with a 24-h culture of the indicator strain, and were further incubated at 37°C for 24 h. The zones of inhibition (in mm) were measured from the edge of the growth of *S*. *suis* to the margin of the inhibitory zone.

### Detection of *suiA* and *sslA* structural genes

The presence in the *S*. *suis* 65 genome of the structural genes *suiA* and *sslA*, coding respectively for suicin 3908 and suicin 90–1330 previously characterized in our laboratory [16, 17], was investigated by PCR. PCR reactions consisted of 41.5 μl of PCR grade water, 5 μl of 10× Taq reaction buffer, 1 μl of nucleotide mix, 0.6 μl each of the appropriate forward and reverse primers for *suiA* (A284: 5’-CAAACTGCAACTGATCAAGAAATTA-3’ and A398R: 5’-AATTTTTGCACCCAGAGATGAATCC-3’, respectively), *sslA* (G48: 5’-AAACAACTCAGGAGCTTCAC-3’ and G130R: 5’-CACAGGTCATCAAAATACCC-3’, respectively), or *gdh* (used as a positive control; GDH645: 5’-TTTGGTTTACTTCACTGATAACATG-3’ and GDH794R: 5’-GAGTCTGAAACAGAAATAACTTTTG-3’, respectively), 1 μl of EconoTaq DNA polymerase (5 U/μl), and 1 μl of genomic DNA as a template. The PCR was performed with a DNA Thermal Cycler 480 (Perkin-Elmer, Waltham, MA, USA) according to the EconoTaq reaction protocol of Lucigen Corporation (Middleton, WI, USA). The reaction was carried out for 30 cycles with the following temperature-time profile: 95°C for 1 min, 50°C for 1 min, and 72°C for 1 min. At the end of the amplification protocol, the samples were incubated at 72°C for 3 min. A 1% agarose gel was used to analyze the PCR products.

### Purification of bacteriocin

One hundred μl of an overnight culture of *S*. *suis* 65 was spread onto twelve THA plates supplemented with 0.01% Tween 80. Following incubation at 37°C under aerobic conditions for 24 h, bacterial cells were removed by scraping the surface of plates, and the solid medium of each plate was chopped into small pieces and transferred into 50-ml polypropylene tubes containing 2 ml of 20 mM 2-(N-morpholino)ethanesulfonic acid (MES) buffer pH 5.5 and 0.01% Tween 80 prior to freezing at -80°C for a minimum of 2 h. Thereafter, the culture medium was thawed at 55°C and the tubes were subjected to centrifugation (10,000 x *g* for 10 min). The liquid phases were recovered and pooled. The above procedure was repeated and all fractions were pooled. Residual bacteria and agar particles in the final bacteriocin-containing fraction were removed by two centrifugations at 10,000 x *g* for 10 min. The sample was then subjected to cationic exchange high-pressure liquid chromatography (HPLC; MonoS 5/50 GL column; GE Healthcare, Baie d’Urfé, QC, Canada) using an ÄKTA Purifier system (GE Healthcare). Elution was performed at a flow rate of 1 ml/min using a linear gradient of KCl from 0 to 0.65 M in the above MES buffer. The active fractions were detected by spotting 5 μl onto the surface of THA plates inoculated with a lawn of *S*. *suis* MGGUS3 used as the indicator strain (spot test plate assay). Following growth (37°C/24 h), positive fractions showing an inhibitory zone were pooled and dialyzed (1,000 Da cut-off) overnight against 0.01% trifluoroacetic acid (TFA) + 10% acetonitrile. The resulting fraction was then subjected to reversed-phase HPLC (SOURCE 15RPC column; GE Healthcare). Elution was performed at a flow rate of 1 ml/min using a linear gradient of acetonitrile from 10 to 90%. The acetonitrile and TFA were removed by a rotary evaporator prior to analyze the fractions for bacteriocin activity using the spot test plate assay as above. The active fractions were pooled and glycerol was added to a final concentration of 15%, prior to aliquot (100 μl) and store at -80°C. Total purified bacteriocin was quantified as arbitrary units, which correspond to the reciprocal of the highest two-fold serial dilution giving a clear inhibitory zone following application of 5 μl of the bacteriocin solution on a lawn of *S*. *suis* MGGUS3.

### SDS-PAGE analysis

The purified bacteriocin was subjected to sodium dodecyl sulfate-polyacrylamide gel electrophoresis (SDS-PAGE) on a 16.5% Tris-Tricine gel (Bio-Rad Laboratories, Mississauga, ON, Canada), fixed (30 min) in 40% methanol-10% acetic acid, and stained with Coomassie brilliant blue G-250. A gel was also fixed in 10% acetic acid—20% propanol (30 min), washed thoroughly in sterile distilled water (5 x 30 min), and bacteriocin activity was detected using an overlay of THB-soft agar inoculated with the indicator strain *S*. *suis* MGGUS3. Nisin A (Sigma-Aldrich Canada Co.) was used as a positive control.

### Characterization of bacteriocin

The susceptibility of the purified bacteriocin to temperature, pH, and enzymatic treatments was investigated using the spot test plate assay described above and *S*. *suis* MGGUS3 as the indicator bacteria. To evaluate temperature stability, the purified bacteriocin was incubated at 45, 70, 100 or 121°C for 15 min prior to determine the antibacterial activity. To investigate the susceptibility to extreme pHs, the bacteriocin solution was adjusted to pH 2 or 11 by using 0.125 N HCl or 0.125 N NaOH, respectively. After 15 min at room temperature, the bacteriocin solution was diluted 1:2 in PBS to neutralize pH, and the antibacterial activity was then determined. Lastly, the proteolytic enzymes trypsin, chymotrypsin, and proteinase K (Sigma-Aldrich Canada Co.), each at a final concentration of 500 μg/ml, were used to evaluate the susceptibility of the bacteriocin to proteolytic cleavage. Following incubation at 37°C for 30 min, samples were treated for 5 min at 68°C to inactivate the enzymes and the bacteriocin activity was determined.

### Amino acid sequencing

The purified bacteriocin was subjected to SDS-PAGE as above and then electroblotted onto a polyvinylidene diofluoride (PVDF) membrane. The bacteriocin band, localized based on the migration of pre-stained molecular weight markers, was excised and transferred into a microtube. Ethanethiol derivatization of post-translationally modified amino acids of the PVDF-blotted bacteriocin was carried out as previously described by Meyer et al. [18] with slight modifications. In an anaerobic chamber, 200 μl of a reaction mixture containing 280 μl methanol, 200 μl of H_2_O, 65 μl 5 M of NaOH and 60 μl of ethanethiol was added. After incubation at 50°C for 1 h, the solution was acidified by adding 66 μl of 70% (v/v) formic acid and the bacteriocin-blotted PVDF membrane was vacuum-dried. The treated bacteriocin was then sent to the SPARC BioCentre (The Hospital for Sick Children, Toronto, ON, Canada) and subjected to Edman degradation using an Applied Biosystems ABI 492 Procise cLC sequencer (Life Technologies Inc., Burlington, ON, Canada).

### Identification of the putative gene cluster encoding suicin 65

Published *S*. *suis* genomes were blasted for the presence of proteins related to the partial amino acid sequence determined above using the NCBI Protein blast web-based platform (blast.ncbi.nlm.nih.gov) [19]. The putative bacteriocin gene cluster that was identified was used to design primers for PCR amplification using genomic DNA extracted from *S*. *suis* 65. The sequences of the forward and reverse primers were: ext84170: 5’- CTCCTGTAAGATGAACTTGT-3’ and ext84641R: 5’-CTTTATATGAGTCTGTCTGT-3’, respectively. The reaction mixture was prepared as above and the reaction was carried out for 25 cycles with the following temperature-time profile: 95°C for 1 min, 45°C for 1 min, and 72°C for 40 sec. At the end of the amplification protocol, the samples were incubated at 72°C for 3 min. The putative suicin 65 locus was then sequenced.

### Multilocus sequence typing of *S*. *suis* 65

Multilocus sequence typing of *S*. *suis* 65 was performed by PCR amplification and DNA sequencing of seven housekeeping genes (*cpn60*, *dpr*, *recA*, *aroA*, *thrA*, *gki*, *mutS*), as previously described [20].

### Antibiotic susceptibility of *S*. *suis* 65

The susceptibility of *S*. *suis* 65 to antibiotics (amoxicillin, ceftiofur, and penicillin G) was determined as follows. Briefly, a 24-h bacterial culture in THB was diluted in fresh broth medium to obtain an optical density at 660 nm (OD_660_) of 0.2. Equal volumes (100 μl) of bacteria and serial dilutions of antibiotics in THB were mixed in the wells of 96-well plates. Wells with no bacteria or no antibiotics were used as controls. Following a 24-h incubation at 37°C, bacterial growth was recorded visually. Minimal inhibitory concentration (MIC) values (μg/ml) were expressed as the lowest concentration at which no growth occurred. The MIC values were determined in three independent experiments.

## Results

**Fig 1:**
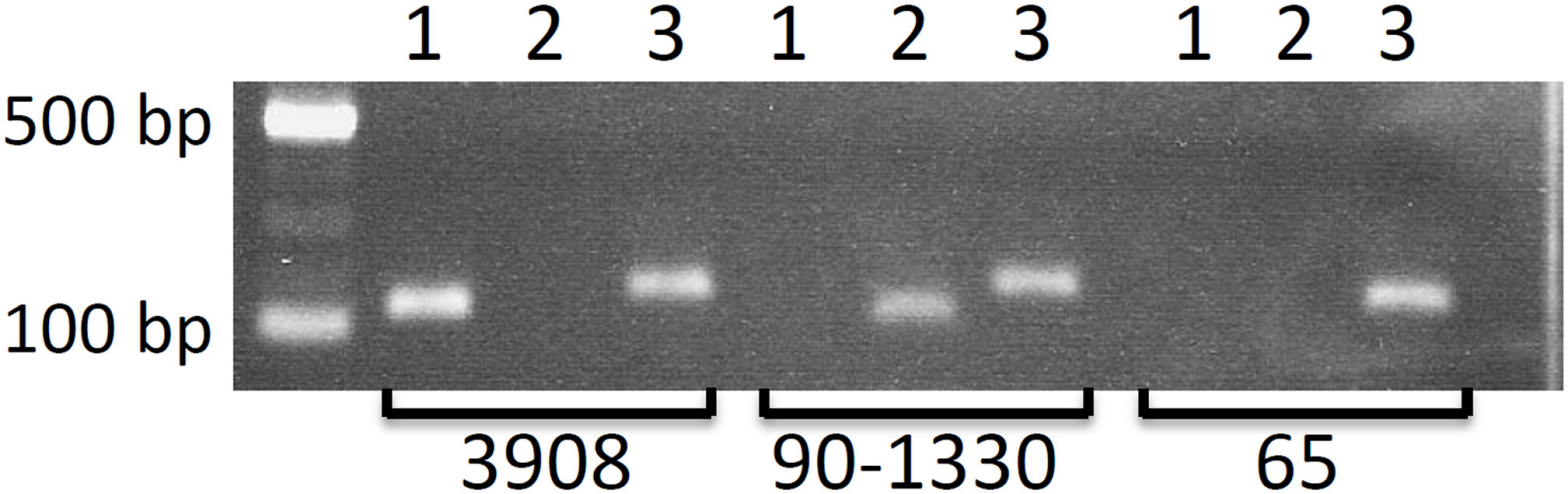
PCR detection of the lantibiotic structural genes *suiA* and *sslA*, and the control gene *gdh* in *S*. *suis* 3908, 90–1330, and 65. Lane 1: *suiA*. Lane 2: *sslA*. Lane 3: *gdh*.

In a previous study [21], *S*. *suis* 65 has been found to exert antagonism against other strains of *S*. *suis* suggesting that it may produce a bacteriocin-like inhibitory substance. We first confirmed the antibacterial activity of *S*. *suis* 65 using the indicator strain *S*. *suis* MGGUS3 in a plate diffusion assay (inhibitory zone = 5.3 ± 0.5 mm). To exclude the possibility that this antibacterial activity resulted from the production of the two previously characterized *S*. *suis* lantibiotics, the presence of the structural genes *suiA* and *sslA*, coding respectively for suicin 3908 and suicin 90–1330, was investigated by PCR. As shown in Fig 1, both genes were absent in *S*. *suis* 65, while *suiA* was identified in *S*. *suis* 3908, and *sslA* in *S*. *suis* 90–1330, thus suggesting that *S*. *suis* 65 produces a novel bacteriocin. The control gene *gdh* was identified in all three *S*. *suis* isolates.

The antibacterial activity of *S*. *suis* 65 was investigated with the diffusion plate assay on additional strains of *S*. *suis*. As reported in Table 1, all strains tested belonging to different STs, isolated in different countries, and showing resistance to antibiotics, including erythromycin and tetracycline, were inhibited to various extents.

**Fig 2:**
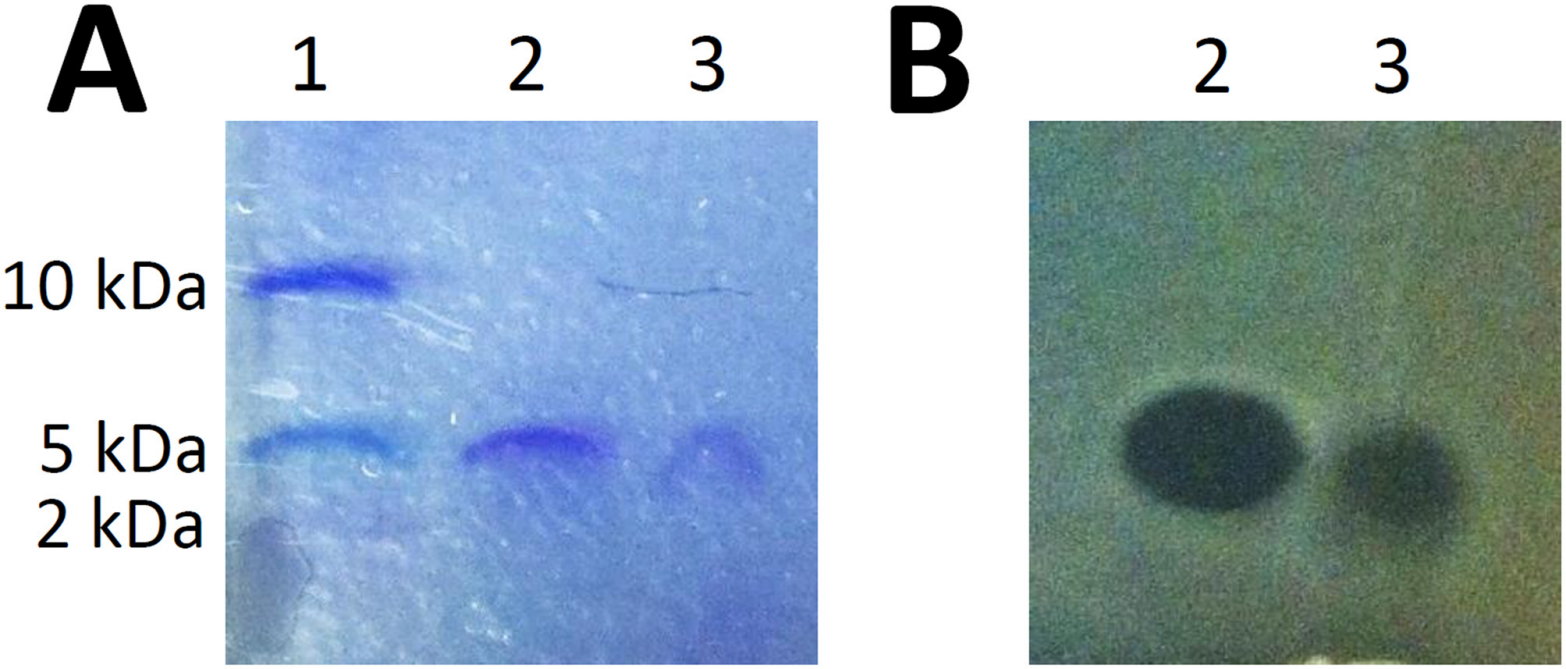
Tris-Tricine SDS-PAGE analysis of the purified bacteriocin produced by *S*. *suis* 65. Panel A. Coomassie blue staining. Panel B. Antibacterial activity detected by an overlay with *S*. *suis* MGGUS3 as the indicator strain. Lane 1: molecular weight markers; Lane 2: commercial nisin A; Lane 3: purified suicin 65.

A preliminary analysis revealed that a 24-h culture supernatant of *S*. *suis* 65 does not possess antibacterial activity thus indicating that the bacteriocin was not secreted following growth in a liquid medium. Consequently, a protocol was developed to prepare a bacteriocin-rich fraction following growth of *S*. *suis* 65 on THA plates. This fraction was then used to purify the bacteriocin to homogeneity by cationic exchange and reversed-phase HPLC. Bacteriocin activity recovered from the reversed-phase chromatography eluted in a single peak and Tricine-SDS-PAGE analysis of the purified bacteriocin yielded a single band stained by Coomassie blue and migrating slightly lower than the commercial lantibiotic nisin A, which has a molecular mass of 3,354 Da (Fig 2A). An overlay with the indicator strain (*S*. *suis* MGGUS3) correlated the bacteriocin activity with the protein band (Fig 2B). From the twelve THA plates of *S*. *suis* 65, the purification protocol allowed the recovery of 8,000 arbitrary units of bacteriocin, as defined in Materials and Methods.

The purified bacteriocin was subjected to various treatments to determine its stability to heat, pH, and proteolytic enzymes (Table 2). Using the spot test assay, the bacteriocin was highly heat stable as the antibacterial activity against the indicator strain (MGGUS3) was still detected even after treatment at 121°C for 15 min. The bacteriocin was also found to be stable under a wide range of pH since antibacterial activity was still observed following exposure at pH 2 and 11. Moreover, the antibacterial activity remained active following a treatment of the purified bacteriocin with proteolytic enzymes (trypsin, chymotrypsin, proteinase K). Lastly, using the spot test assay, the purified bacteriocin was found to be active against all strains that have been tested in the plate diffusion assay with the producing strain.

**Table 2:** Stability of the purified suicin 65 determined using the spot test assay and *S*. *suis* MGGUS3 as the indicator strain.

Treatment	Inhibitory activity
45°C / 15 min	+
70°C / 15 min	+
100°C / 15 min	+
121°C / 15 min	+
pH 2 / 15 min	+
pH 11 / 15 min	+
Trypsin / 30 min	+
Chymotrypsin / 30 min	+
Proteinase K / 30 min	+

**Fig 3:**
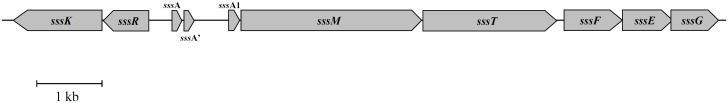
Genetic organization of the putative suicin 65 gene cluster.

Peptide sequencing of the purified bacteriocin was carried out by Edman degradation. The first nine amino acids yielded the following sequence: Gly_1_-Lys_2_-Asn_3_-Gly_4_-Val_5_-Phe_6_-Lys_7_-X(Ser or Cys or Thr)_8_-Ile_9_. Residue 8 yielded a peak close to the Phe peak, accompanied by a relatively strong Leu signal, which is characteristic of Thr residues modified to either dehydrobutyrine (Dhb) or a methyllanthionine (MeLan) moiety [18]. Using the NCBI Protein blast web-based platform, published genomes of *S*. *suis* were analyzed for the presence of a protein showing similarity with the above 9-amino acid sequence. This analysis led to the identification of two genes (ssuisDraft_3894 and ssuisDraft_3893), annotated as type A lantibiotic, in the genome of strain *S*. *suis* 89–1591 (serotype 2, ST25) that were contiguous and shared 92% identity of residues (nucleotides or amino acid). Further analysis of the 89–1591 genome revealed the presence of a complete lantibiotic biosynthesis locus. No additional locus that may correspond to suicin 90–1330 or suicin 3908 were found. Based on this gene locus identified in strain 89–1591, primers were designed for PCR amplification in *S*. *suis* 65. Following sequencing, the complete *sss* (*streptococcus suis suicin*) gene cluster of *S*. *suis* 65 was identified and was found to contain ten open reading frames. As reported in Fig 3, the locus encodes the suicin 65 precursors (*sssA*, *sssA’*), a synthetase involved in lantibiotic modification (*sssM*), an ABC transporter (*sssT*), a response regulator (*sssR*), a sensor histidine kinase (*sssK*), and three immunity proteins (*sssF*, *sssE*, *sssG*). A small open reading frame (*sssA1*), for which no functions has been assigned yet, was located upstream the *sssM* gene.

**Fig 4:**
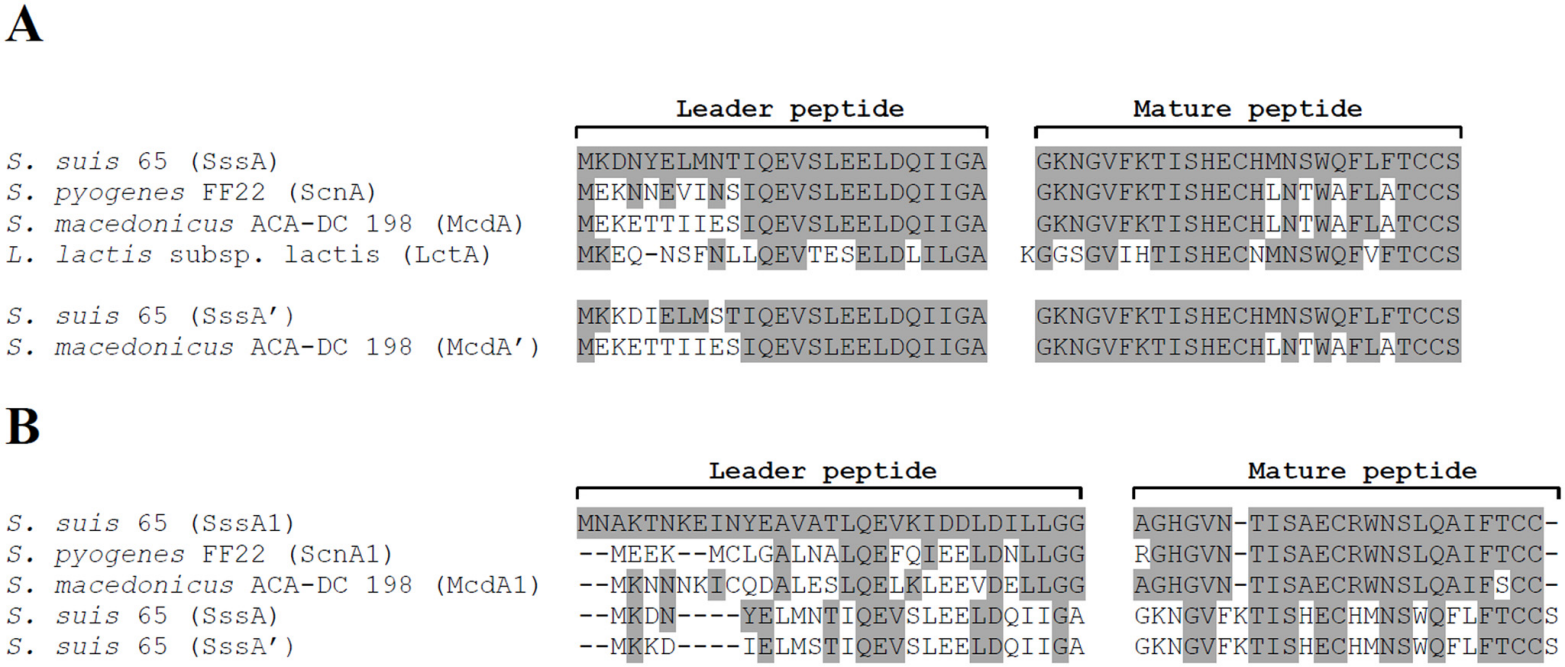
Comparison of the amino acid sequence of suicin 65 with other lantibiotics produced by *S*. *macedonicus* ACA-DC 198 (macedocin), *S*. *pyogenes* FF22 (streptococcin), and *L*. *lactis* subsp. *lactis* 481 (lacticin). The left and right blocks of the sequence refer to the leader and mature peptides, respectively. Panel A: SuiA and SuiA’ and their homologues; Panel B: SuiA1 and its homologues.

From the structural genes *sssA* and *sssA’*, the inferred amino acid sequence of the leader peptide contains 25 amino acids while that of mature unmodified peptide consists of 26 amino acids (Fig 4). The deduced molecular mass of the unmodified mature bacteriocin is 3,005 Da. Comparison of the SssA amino acid sequence with previously characterized lantibiotics highlighted high levels of identity with streptococcin FF22 (80%) produced by *S*. *pyogenes* FF22, macedocin ACA-DC 198 (75%) produced by *S*. *macedonicus* ACA-DC 198, and lacticin 481 (62%) produced by *Lactococcus lactis* subsp. *lactis* 481 (Table 3). Fig 4 indicates that the mature peptides of *S*. *pyogenes* FF22 and *S*. *macedonicus* ACA-DC 198 lantibiotics differ from that of suicin 65 in only four amino acids. In regard to the duplicate structural gene *sssA’*, the inferred amino acid sequence of the leader peptide and the mature peptide showed respectively 92% and 100% of identity with the corresponding peptides encoded by the sssA gene. Regarding nucleic acid residues, *sssA* and *sssA’* genes related to mature peptide showed 86.4% identity. The macedocin ACA-DC 198 locus in *S*. *macedonicus* also contains a duplicate structural gene *mcdA’* that showed 76% of identity (amino acid sequence) with *sssA’* (Fig 4). Lastly, the gene *sssA1* whose function in the *sss* locus is not identified yet showed poor homology (65%) in amino acid sequence with its homologues *scnA1* and *mcdA1* found in *S*. *pyogenes* and *S*. *macedonicus*, respectively (Fig 4).

**Table 3:** Percentage identity in deduced amino acid sequences between the products of the *S*. *suis* suicin 65 gene cluster and the gene cluster bacteriocins of *S*. *macedonicus* ACA-DC 198, *S*. *pyogenes* FF22, *L*. *lactis* subsp. *lactis* 481 and *S*. *suis* 89–1591.

Genes	% identity			
	*S*. *macedonicus* ACA-DC 198	*S*. *pyogenes* FF22	*L*. *lactis* subsp. *lactis* 481	*S*. *suis* 89–1591
*K*	73	78	-	100
*R*	82	86	-	100
*A*	75	80	62	100
*A’*	76	-	-	100
*A1*	65	65	-	-^1^
*M*	62	62	30	-^1^
*T*	72	74	39	100^1^
*F*	82	81	44	99
*E*	34	33	22	100
*G*	49	44	26	100

^1^The complete genes *A1* and *M*, as well as half of the gene *T* are deleted

Table 3 reports the percentage identity of *S*. *suis* 65 genes of the lantibiotic biosynthesis locus with the corresponding genes found in *S*. *macedonicus* ACA-DC 198, *S*. *pyogenes* FF22, *L*. *lactis* subsp. *lactis* 481, and *S*. *suis* 89–1591. The genes *E* and *G* coding for immunity proteins showed the lowest percentage identity. As in the lantibiotic locus of *S*. *suis* 65, the lantibiotic locus of *S*. *macedonicus* also contains a duplicate of the structural gene (*A and A’*) as well as a non-related gene (*A1*). The lantibiotic locus of *S*. *suis* 89–1591 has a 4,473 bp deletion that results in the lack of the gene *A1*, the gene *M*, and half of the gene *T*. These observations suggest that active bacteriocin may not be produced by this strain. Using the plate diffusion assay, it was confirmed that *S*. *suis* 89–1591 does not possess antibacterial activity against the indicator strain *S*. *suis* MGGUS3 (data not shown). Moreover, *S*. *suis* 89–1591 did not possess immunity against the bacteriocin produced by *S*. *suis* 65 (Table 1).

**Fig 5:**
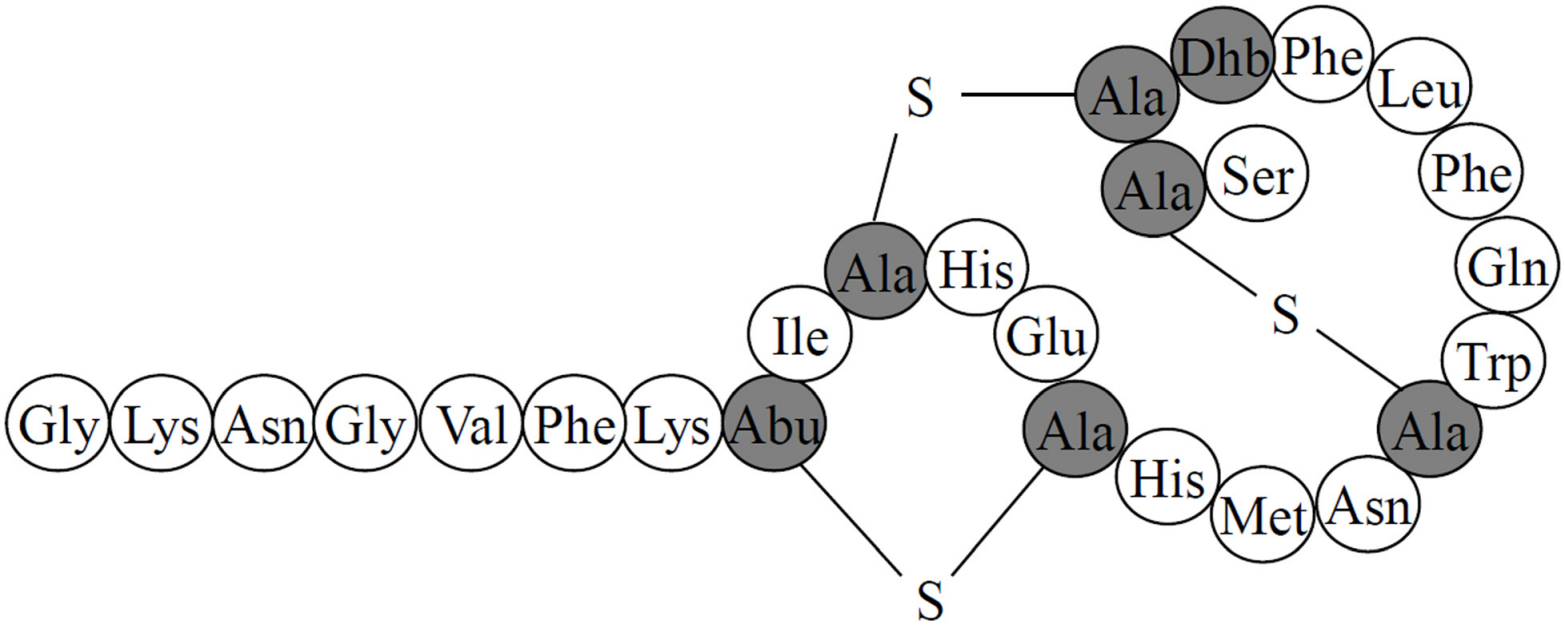
Proposed structure of suicin 65. Mature peptide where Ser and Thr residues which are posttranslationally dehydrated to dehydrobutyrine (Dhb), or involved in the formation of lanthionine (Ala-S-Ala) and methyllanthionine (aminobutyrate[Abu]-S-Ala), respectively, with cysteine residues, are shaded in grey.

Fig 5 presents the proposed structure of suicin 65 based on the deducted amino acid sequence of the structural genes *sssA* and *sssA’* and previously reported structures of lantibiotics with high identity with suicin 65. It has a linear part in N-terminal and a globular part in C-terminal. Moreover, it shows the presence of two lanthionine groups (Ala-S-Ala) between residues 17 and 25 and 10 and 24, and one methyllanthionine group (Aminobutyrate-S-Ala) between residue 8 and 13, and a dehydrobutyrine (Dhb) in residue 23.

Lastly, given that *S*. *suis* 65 may have potential probiotic and protective applications, it was of interest to perform MLST to determine to what sequence type (ST) it belongs. This analysis revealed that *S*. *suis* 65 is an ST28, that includes strains with a low or no virulence in a mouse model [20]. We also evaluated the susceptibility of *S*. *suis* 65 to antibiotics. *S*. *suis* 65 was found to be highly sensitive to amoxicillin with a MIC of 0.0049 μg/ml as well as to penicillin G and ceftiofur with a MIC of 0.0098 μg/ml.

## Discussion

Antibiotics are widely used in animal agriculture to treat and/or prevent bacterial diseases in order to reduce economic losses. Moreover, with the objective to maintain health and increase productivity, animal production is sometimes associated with the regular use of antibiotics, thus promoting the emergence of antibiotic-resistant bacteria. In the United States, it has been estimated that ~80% of the nation’s annual antimicrobial consumption accounts for livestock production [22]. One strategy to overcome the fact that bacterial pathogens become increasingly resistant to several antibiotics is to look for new antimicrobial agents or alternative therapeutic strategies. In this regard, bacteriocins, which are ribosomally synthesized antimicrobial peptides of bacterial origin, have been proposed to represent a promising option to antibiotics in livestock production [9, 10]. Recently, our laboratory purified and characterized two bacteriocins of the lantibiotic family produced by *S*. *suis* and active against this swine pathogen [16, 17]. In this study, we report the purification and characterization of a novel class I type B lantibiotic produced by *S*. *suis* 65.

While the two previously reported *S*. *suis* lantibiotics (suicin 90–1330 and suicin 3908) were purified from a bacterial culture supernatant, the one produced by *S*. *suis* 65 was not detected in broth media and consequently has been purified from solid culture media. Lantibiotics produced by *Streptococcus mutans* [23] and *Lactobacillus plantarum* [24] were also reported to be only produced following growth on solid media. As observed for bacteriocins of the lantibiotic family, the purified suicin 65 was found to be stable to heat, pH and proteases. The bacteriocin produced by *S*. *suis* 65 was found to be active against all *S*. *suis* strains tested, including strains possessing resistance to erythromycin and/or tetracycline.

Suicin 65 is a class I type B lantibiotic made of 26 amino acid residues (mature peptide) with a linear portion in N-terminal and a globular portion in C-terminal. The previously reported suicin 3908 produced by *S*. *suis* 3908 was also a class I type B lantibiotic (33 amino acid residues); however, the two bacteriocins exibit a poor identity (20.6%) for the unmodified mature peptide. Mature unmodified suicin 65 showed a high homology with macedocin ACA-DC 198 (84.6%), streptococcin FF22 (84.6%), and lacticin 481 (74.1%) produced by *S*. *macedonicus* [25], *S*. *pyogenes* [26], and *L*. *lactis* subsp. *lactis* [27], respectively.

A genetic locus containing ten open reading frames has been identified in *S*. *suis* 65 and is most likely responsible for suicin 65 regulation, biosynthesis, and immunity, although a direct proof for its involvement would be to correlate an absence of activity with a mutation created in the structural gene. Surprisingly, the locus shows the presence of a duplicate of the structural gene. More specifically, immediately downstream of the *sssA* structural gene, there is another open reading frame named *sssA’*. The mature peptides of *sssA* and *sssA’* possess a 100% homology. Analysis by RT-PCR provided evidence that both structural genes are cotranscribed and part of the same operon (data not shown). The presence of duplicate structural genes, also cotranscribed, has been reported in the genetic locus of macedocin ACA-DC 198 [25]. In addition, the *sss* genetic locus showed the presence of a third structural gene (*sssA1*) that appears unrelated to suicin 65 and that exhibit 65% homology with similar genes found in the genetic locus of macedocin ACA-DC 198 [25] and streptococcin FF22 [26]. Further studies are required to determine whether the peptide encoded by *suiA1* corresponds to a functional lantibiotic and how its expression is regulated in *S*. *suis* 65.

Most of the *S*. *suis* serotype 2 strains from North America have been found to belong to ST25 or ST28, which are significantly less virulent than ST1 strains [20]. More specifically, ST28 isolates are essentially avirulent in a mouse model, while ST25 strains are moderately virulent and able to induce severe infections [20]. MLST performed on *S*. *suis* 65 showed that it is an ST28 isolate. Moreover, *S*. *suis* 65, which has been isolated from tonsils of a healthy pig, did not induce clinical signs when inoculated in pathogen-free piglets [28]. We also showed that this strain is highly susceptible to antibiotics (amoxicillin, ceftiofur, penicillin G) currently used in the swine industry. Taken together, these characteristics suggest that *S*. *suis* 65 may be safe, although additional animal studies will have to be carried out to confirm this. Future research should evaluate whether the suicin 65 or the producing strain *S*. *suis* 65 could be used as a therapeutic agent targeting *S*. *suis*.
